# Intra-Tumoral Secondary Follicle-like Tertiary Lymphoid Structures Are Associated with a Superior Prognosis of Overall Survival of Perihilar Cholangiocarcinoma

**DOI:** 10.3390/cancers14246107

**Published:** 2022-12-12

**Authors:** Fa-Peng Zhang, Ke Zhu, Tai-Feng Zhu, Chao-Qun Liu, Hong-Hua Zhang, Lei-Bo Xu, Gang Xiao, Chao Liu

**Affiliations:** 1 Department of Biliary-Pancreatic Surgery, Sun Yat-Sen Memorial Hospital, Sun Yat-Sen University, Guangzhou 510120, China; 2 Guangdong Provincial Key Laboratory of Malignant Tumor Epigenetics and Gene Regulation, Guangdong-Hong Kong Joint Laboratory for RNA Medicine, Department of Biliary-Pancreatic Surgery, Sun Yat-Sen Memorial Hospital, Sun Yat-Sen University, Guangzhou 510120, China; 3 Department of Pathology, Guangdong Provincial People’s Hospital, Academy of Medical Sciences, Guangzhou 510080, China; 4 Department of Thoracic Surgery, Guangzhou First People’s Hospital, South China University of Technology, Guangzhou 510180, China

**Keywords:** perihilar cholangiocarcinoma, tertiary lymphoid structures, overall survival, recurrence, immunity

## Abstract

**Simple Summary:**

Tertiary lymphoid structures (TLSs) are reported to play an antitumor role in a variety of tumors and are a predictive marker of immunotherapy efficacy and survival outcomes but have not been reported in perihilar cholangiocarcinoma (pCCA). In the present study, hematoxylin and eosin, immunohistochemical staining and multiplex immunofluorescence were performed on tissue sections from 93 pCCA patients to analyze the maturity and number of intra-tumoral TLSs and their relevance to clinical characteristics, overall survival (OS), and recurrence-free survival. The results suggest that intra-tumoral mature TLSs, or secondary TLSs (S-TLSs), significantly improve OS in pCCA patients, suggesting that S-TLSs contribute to effective antitumor immunity and hold promise for diagnostic and therapeutic development in pCCA.

**Abstract:**

Ectopic lymphoid structures termed tertiary lymphoid structures (TLSs) have an immunomodulatory function and positively affect prognosis in certain cancers. However, their clinical relevance and prognostic utility in perihilar cholangiocarcinoma (pCCA) are unknown. Therefore, determining the involvement and prognostic utility of TLSs in pCCA is the aim of this study. Ninety-three patients with surgically resected pCCA were included retrospectively. Hematoxylin and eosin and immunohistochemical staining identified and classified the TLSs, and multiplex immunofluorescence determined the TLS composition in the pCCA sample. The correlations between clinical features and TLSs were analyzed using either Fisher’s exact test or the Chi-squared test. Recurrence-free survival (RFS) and overall survival (OS) correlations with TLSs were analyzed using Cox regression and Kaplan–Meier analyses. We identified TLSs in 86% of patients with pCCA, including lymphoid aggregates (6.45%), primary (13.98%) and secondary follicles (65.59%). Patients with intra-tumoral secondary follicle-like TLSs (S-TLSs) had better OS (*p* = 0.003) and RFS (*p* = 0.0313). The multivariate analysis identified the presence of S-TLSs as a good independent prognostic indicator for OS but not for RFS. Interestingly, the presence of S-TLS only indicated better 5-year OS in 54 patients without lymph node metastasis (LNM^−^, *p* = 0.0232) but not in the 39 patients with lymph node metastasis (LNM^+^, *p* = 0.1244). Intra-tumoral S-TLSs predicted longer OS in patients with surgically resected pCCA, suggesting intra-tumoral S-TLSs’ contribution to effective antitumor immunity and that S-TLSs hold promise for diagnostic and therapeutic development in pCCA.

## 1. Introduction

Cholangiocarcinoma (CCA) is the second most common primary liver malignancy after hepatocellular carcinoma [1]. Based on anatomical boundaries, CCA is further categorized as distal cholangiocarcinoma (dCCA), perihilar cholangiocarcinoma (pCCA), and intrahepatic cholangiocarcinoma (iCCA). Among them, pCCA accounts for more than half of the cases and has a worse prognosis than iCCA [2]. Current therapies used to treat pCCA suffer from poor efficacy, and the only options to cure pCCA are radical surgery or liver transplantation [3,4,5]; however, the rate of postoperative recurrence is very high (75% of patients) [6,7,8]. The fate of the patient depends on the conventional prognostic factors used in clinical practice, such as tumor stage and lymph node status. In many cases, these factors are insufficient to distinguish between tumors at the same clinical stage, which might have distinct clinical outcomes and respond differently to the same treatment. Thus, considering pCCA’s poor prognosis, the identification of efficacious prognostic biomarkers is urgently required, which might be used to formulate individual treatment strategies according to different situations.

The complex interactions between the host immune response and tumor cells have become a research hotspot. In particular, how is a patient’s clinical outcome affected by infiltrating immune cells [9]? Recently, immunotherapy has been considered a promising radical treatment for tumors, in addition to radical resection. Immune checkpoint molecules, such as programmed cell death 1 ligand 1 (PD-L1), cytotoxic T-lymphocyte associated protein 4 (CTLA4), HERV-H LTR-associating 2 (HHLA2), and fibrinogen Like 1 (FGL1), can indicate the prognosis and predict the effect of immunotherapy in different tumors [10,11,12]. Recent data from a phase III randomized, double-blind placebo-controlled, multi-regional, international TOPAZ-1 clinical trial (Retrieved 6 December 2022, from ClinicalTrial.gov, NCT03875235) in advanced biliary tract cancer showed that durvalumab in combination with gemcitabine plus cisplatin significantly extended survival compared to gemcitabine plus cisplatin alone [13]. This demonstrates the potential of immunotherapy for CCA. The presence and activation of various immune cells (e.g., CD4^+^ T cells, CD8^+^ T cells, and B cells) are required for successful antitumor immunity [14,15,16]. This notion is supported by the presence of well-organized clusters of tumor-infiltrating lymphocytes, termed intra-tumoral tertiary lymphoid structures (TLSs), which might induce an advanced immune response [9]. Recently, scholars have investigated the significance of TLSs and have shown that the presence of TLSs indicated better prognosis and immunotherapeutic efficacy [17]. Therefore, TLSs are expected to become important markers to guide tumor immunotherapy.

TLSs comprise ectopic aggregates of lymphoid cells that occur in tumor tissues or sites of chronic inflammation. They include a B-cell follicle enveloped by a T-cell zone containing a variety of T cells and dendritic cells (DCs) and high endothelial venules (HEV) [18,19]. TLSs generate plasma cells, central memory T cells, and effector T cells, which are believed to have vital functions in the anti-tumor immune response [17,20,21]. Despite the presence of TLSs being reported to be associated with better prognosis and reduced recurrence risk in almost all types of solid tumors [22,23,24,25,26], a few studies suggested that TLSs are a poor prognostic indicator [27,28,29]. Interestingly, different groups reported opposite prognostic effects of TLSs in hepatocellular carcinoma (HCC), possibly because of the different models used [24,27]. In iCCA, the function of TLSs was recently reported [30]. However, their role in pCCA remains unclear.

To bridge this knowledge gap, in the current study, we recruited 93 patients with pCCA who received surgical resection to explore the prognostic significance of TLSs. We established that the presence of secondary follicle-like TLSs (S-TLSs) is a positive prognostic factor for patients with pCCA, suggesting that S-TLSs might have an anti-tumor immune function. Determining the mechanism by which S-TLSs are formed in pCCA could guide the development and implementation of immunotherapeutic interventions.

## 2. Materials and Methods

### 2.1. Patients and Samples

We selected 93 patients with pCCA, which were verified histologically from January 2012 to December 2019, from the records of the Sun Yat-sen Memorial Hospital, Sun Yat-sen University. The following inclusion criteria were used: (1) complete clinical and follow-up data; (2) available histological slides and paraffin-embedded blocks; (3) unequivocal diagnosis of pCCA and had undergone surgical resection; and (4) lack of prior antitumor therapy. We extracted the patients’ clinicopathological and demographic characteristics from the electronic medical records. The 8th edition of the American Joint Committee on Cancer (AJCC^8th^) cancer staging manual was used to determine the tumor-node-metastasis (TNM) staging of pCCA [31]. The period between surgery to the first instance of recurrence or the last date of follow-up represented recurrence-free survival (RFS). The period between surgery and death or the last date of follow-up defined overall survival (OS). The patients were subjected to follow-up every 3 to 6 months, which comprised computed tomography, laboratory tests (including tumor markers), and physical examinations. Radiological tumor progression, rather than increasing tumor markers alone, confirmed recurrence. As required, biopsy sampling, 18-fluorodeoxyglucose positron emission tomography (PET) scans and magnetic resonance imaging (MRI) were also carried out. pCCA tissues were obtained from the Sun Yat-sen Memorial Hospital, with signed informed consent from patients and ethical committee approval.

### 2.2. Immunohistochemistry (IHC) Staining

IHC staining was carried out on 4 μm-sections of formalin-fixed paraffin-embedded (FFPE) tissue obtained from patients with pCCA. Xylene was used to deparaffinize the sections, which were then subjected to hydration using an ethanol series. Epitope retrieval was accomplished by boiling the sections in citrate buffer (pH 6.0) or Tris-EDTA (pH 9.0) under high pressure for 10 min and then leaving them to self-cool. Endogenous peroxidase was quenched for 10 min using 3% hydrogen peroxide, followed by blocking of nonspecific binding by incubation for 30 min in 5% bovine serum albumin (BSA). The sections were then incubated with primary antibodies: rabbit anti-CD20 (ab78237, Abcam, Cambridge, UK), rabbit anti-CD21 (ab75985, Abcam, Cambridge, UK) overnight at 4 °C. After washing, the sections were incubated with biotin-labeled secondary antibodies (K5007, Dako at Agilent, Santa Clara, CA, USA) at 37 °C for 30 min. Finally, the sections were washed with phosphate-buffered saline (PBS) and stained for 3–10 min using diaminobenzidine (DAB) solution according to which primary antibody was used, followed by counterstaining using hematoxylin. The whole slides were scanned using a Vectra^®^ Polaris™ instrument (Akoya Biosciences, Menlo Park, CA, USA).

### 2.3. Multiplex Immunofluorescence Assay

For the TLS multiplex immunofluorescence assay, a 5-color fluorescent IHC Kit (abs50013, Absin, Shanghai, China) was used to stain 4 μm-thick FFPE tissue sections. First, all the slides were deparaffinized and the antigen was retrieved as described in “Immunohistochemistry (IHC) staining”. Thereafter, the sections were exposed to primary antibodies (including those recognizing CD20, CD21, CD8, and protein N-terminal asparagine amidase (PNAD)) for 1 h at room temperature, to secondary antibodies conjugated to horseradish peroxidase (HRP) for 10 min at room temperature, and to Tyramide signal amplification (TSA) dye for 10 min at room temperature. Subsequent rounds of staining followed the same procedure, starting with antigen retrieval and ending with TSA dying. We used the following fluorophores for detection: CD20-TSA dye 520, CD21-TSA dye 570, CD8-TSA dye 620, and PNAD-TSA dye 700. Nuclear counterstaining was achieved using 4′,6-diamidino-2-phenylindole (DAPI). Antifade Reagent (abs50013, Absin, Shanghai, China) was used to mount the slides. The immunofluorescence (IF) images were captured and the markers of interest were identified using a Pannoramic DESK (P-MIDI, P250) and Pannoramic Scanner C.V.2.4 software (3DHISTECH Ltd., Budapest, Hungary).

### 2.4. Hematoxylin and Eosin (H&E) Staining

FFPE tissue sections were subjected to hematoxylin sainting, rinsed under running tap water, restained using eosin Y, dehydrated, cleared, mounted on slides, and scanned using the Vectra^®^ Polaris™ instrument.

### 2.5. TLS Quantification

H&E, CD20, and CD21 staining of paraffin sections were performed to visualize the presence of TLSs in pCCA. TLSs were classified into three types based on their size, cellular composition, and degree of organization: (1) Lymphoid aggregates (Agg): vague, poorly defined lymphocyte clusters (containing at least 20 CD20^+^ cells) but lacking a CD21 signal; (2) Primary follicle-like TLSs (P-TLSs): immature TLSs comprising round clusters of lymphocytes without germinal centers (GCs) (highly dense lymphocytic aggregates with a CD20 signal, but no CD21 signal); and (3) Secondary follicle-like TLSs (S-TLSs): mature TLSs, round clusters of lymphocytes with GCs (high dense lymphocytic aggregates with CD20 and CD21 signals) [24,32,33]. The GCs were identified by the localization of CD21^+^ follicular dendritic cells (FDCs), as described previously [32].

Our review of the cases revealed various types of TLSs, which were classified as TLS^+^ tumors if they contained at least 1 intra-tumoral TLS, and as TLS^−^ tumors if they lacked a TLS. The type of TLS was used to further classify the cases: (1) Agg pCCA: tumors with only Agg, and no P-TLS and S-TLS; (2) P-TLS pCCA: tumors comprising at least one P-TLS, without an S-TLS, with or without Agg; and (3) S-TLS pCCA: tumors comprising at least one S-TLS, irrespective of whether Agg and P-TLS were present. TLSs were identified and quantified based on the whole slides, which were reviewed independently by two observers for the presence and maturity of the TLSs.

### 2.6. Statistical Analysis

SPSS software (22.0, IBM Corp., Armonk, NY, USA) was used to carry out all the statistical analyses. Fisher’s exact test or a Chi-squared test was used to compare the categorical variables. The Kaplan–Meier method was used to plot the survival curves and the log-rank test was used to compare them. The Cox proportional hazards model was used to determine the OS and RFS-related independent prognostic factors. Statistical significance was indicated by a *p*-value less than 0.05.

## 3. Results

### 3.1. Clinicopathological Features of the Patients

For the 93 patients with perihilar cholangiocarcinoma (pCCA) who underwent surgical resection analyzed in this study, we retrieved the following clinical and biological features: age (≤ or >60 years), sex, tumor size (≤ or >2.5 cm), tumor differentiation degree, T staging, lymph node metastasis (LNM) status, M staging, AJCC^8th^ TNM staging, preoperative serum carcinoembryonic antigen (CEA) (≤ or >4 ng/mL) value, carbohydrate antigen 19-9 (CA19-9) (≤ or >135 U/mL) value, HBV infection, and microscopic residual tumor (R0 or R1). The patients’ characteristics are summarized in Table 1.

### 3.2. TLSs in Human pCCA Tissue Specimens

**Figure 1:**
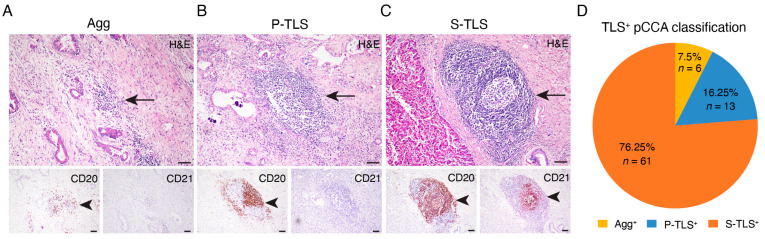
Classification of TLS and TLS-positive patients with pCCA. (**A**–**C**) Representative images of lymphoid aggregates (Agg, left panel), primary follicle-like TLS (P-TLS, middle panel), and secondary follicle-like TLS (S-TLS, right panel) in human pCCA specimens as assessed using IHC staining for CD20 and CD21 and H&E staining. (**D**) Pie charts indicating the TLS composition of patients with pCCA. Scale bar in (**A**–**C**) represents 200 μm. The black arrowheads indicating TLSs in the below IHC staining image of CD20 and CD21 match the black arrows in the continuous section H&E staining image above.

The pathological H&E, CD20, and CD21 immunohistochemical staining identified TLSs in 80 pCCA samples (86.0%) (Table 1). Among the TLS^+^ pCCA samples, six cases (7.5%) were Agg-type TLSs, 13 cases (16.25%) were P-TLSs, and 61 cases (76.25%) were S-TLSs (Figure 1).

**Figure 2:**
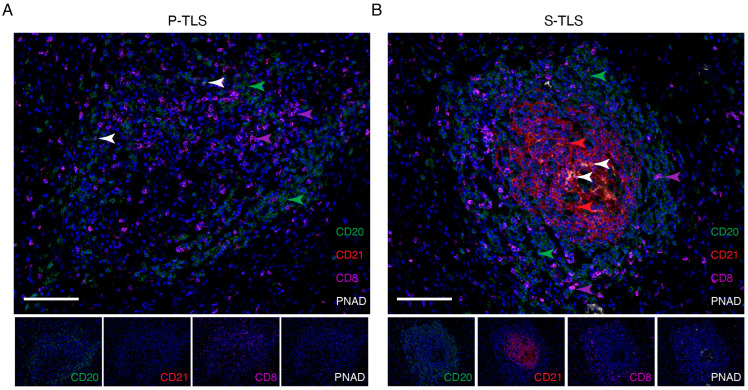
Representative images of multiplex immunofluorescence staining showing the cellular composition of primary and secondary TLSs in pCCA. CD20, CD21, CD8, PNAD, and DAPI were labeled as B cells (green), follicular dendritic cells (red), CD8^+^ T cells (pink), high endothelial venules (white), and nuclei (blue) in P-TLSs (**A**) and S-TLSs (**B**), respectively. The scale bar represents 100 μm.

The results of multiplex immunohistochemistry (mIHC) showed that CD20^+^ B cells, PNAD^+^ HEV, and CD8^+^ T cells could be observed in P-TLSs and S-TLSs. GCs were identified in S-TLSs but not in P-TLSs by the presence of CD21^+^ FDCs (Figure 2). These results demonstrated major cell distribution in the various types of TLSs, suggesting complex interactions among different cell types.

### 3.3. Correlations between Clinicopathological Features and TLSs

Apart from an association with the T stage (*p* = 0.008) and the TNM stage (*p* = 0.036), TLS^+^ pCCAs did not correlate with any other pathological, biological, or clinical feature (Table 1). Furthermore, the correlation between TLS maturity and clinicopathological characteristics was explored. The results demonstrated no correlation between Agg status and any clinical or biological variable (Supplementary Table S1), while the presence of P-TLSs correlated negatively with the T stage (Supplementary Table S2), and the presence of S-TLSs correlated positively with patient age and negatively with the presence of the LNM and TNM stages (Table 2).

### 3.4. TLSs Signify a Favorable Prognosis in pCCA

**Figure 3:**
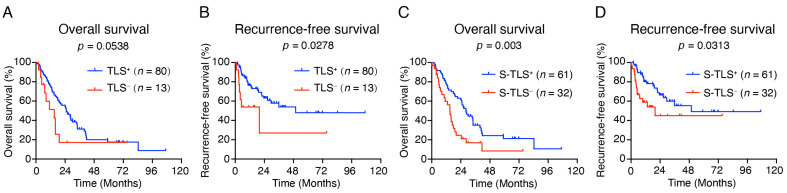
Impact of TLSs on OS and RFS in patients with pCCA. (**A**,**B**) Kaplan–Meier curves show the comparison of OS ((**A**), *p* = 0.0538) and RFS ((**B**), *p* = 0.0278) in patients with pCCA according to the presence or absence of TLSs. (**C**,**D**) Kaplan–Meier curves show the comparison of OS ((**C**), *p* = 0.003) and RFS ((**D**), *p* = 0.0313) in pCCA patients according to the presence or absence of S-TLSs.

Next, the prognostic significance of TLS in human pCCA was assessed. According to Kaplan–Meier survival analysis, the presence of TLSs correlated with a good RFS outcome but not with OS (Figure 3A,B). Previous studies have shown that mature TLSs can be used as prognosticators for OS and RFS in lung squamous cell carcinoma and colorectal cancer [33,34]. Thus, we analyzed the prognostic impact of TLSs with different levels of maturity using log-rank tests. Kaplan–Meier survival analysis showed that the median survival was significantly improved in the S-TLS^+^ group compared with those in the TLS^−^, Agg^+^, and P-TLS^+^ groups (Supplementary Figure S1A,B). In addition, only the presence of S-TLSs improved the OS and RFS but not Agg or P-TLSs (Figure 3C–D and Supplementary Figure S1C–F). These results demonstrated that S-TLSs were a better prognosticator for OS and RFS than Aggs and P-TLSs.

To investigate whether S-TLSs could be used as an independent prognostic indicator in pCCA, univariate and multivariate Cox regression analyses were carried out. In the univariate analysis, lymph node metastasis positive (hazard ratio (HR) = 1.87; 95% confidence interval (CI), 1.151–3.038; *p* = 0.012), elevated value of serum CEA (>4 ng/mL; HR = 1.755; 95% CI, 1.092–2.833; *p* = 0.021), and increased value of CA19-9 (>135 U/mL; HR = 1.850; 95% CI, 1.112–3.049; *p* = 0.016) were associated with increased risk of overall survival, while the presence of S-TLSs (HR = 0.472; 95% CI, 0.284–0.784; *p* = 0.004) was the only variable that correlated with reduced risk (Table 3). Recurrence correlated significantly and positively with sex (male; HR = 0.433; 95% CI, 0.221–0.848; *p* = 0.015), raised serum CEA (>4 ng/mL; HR = 2.257; 95% CI, 1.155–4.412; *p* = 0.017), and elevated serum CA19-9 (>135 U/mL; HR = 2.806; 95% CI, 1.223–6.44; *p* = 0.015). Meanwhile, the presence of S-TLS (HR = 0.477; 95% CI, 0.24–0.949; *p* = 0.035) indicated a favorable prognosis (Supplementary Table S3). In the multivariate analysis, only the presence of S-TLSs (HR = 0.570; 95% CI, 0.330–0.985; *p* = 0.044) was identified as independent prognostic factors for OS but not for recurrence (Table 3 and Supplementary Table S3).

### 3.5. S-TLS Correlates with Low Incidence of Lymph Node Metastasis in pCCA

**Figure 4:**
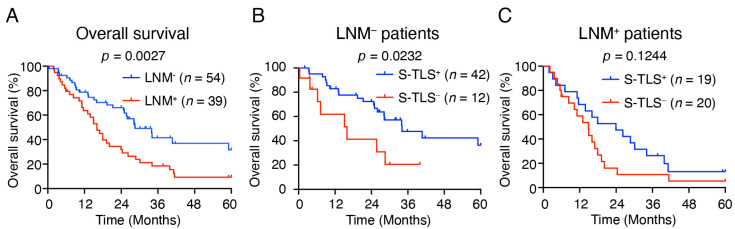
The relationship between S-TLS and Lymph node metastasis. (**A**) Kaplan–Meier curves show the comparison of OS in patients with pCCA according to the presence or absence of lymph node metastasis. (**B**,**C**) Kaplan–Meier curves show the comparison of OS in pCCA patients with or without lymph node metastasis according to the presence or absence of S-TLSs.

Lymph node metastasis (LNM) is considered to be an important risk factor for the overall survival of pCCA [35,36]. We then assessed whether the prognostic effect on OS of S-TLS was influenced by lymph node metastasis status. Consistent with previous studies [8,37], our cohort showed that patients with lymph node metastasis (LNM^+^ patients) had a worse OS (*p* = 0.0027, Figure 4A) and S-TLS^+^ pCCA patients had a lower rate of lymph node metastasis (Table 2, 31.1% vs. 62.5%). In addition, we used lymph node metastasis status as a hierarchical variable to evaluate the prognostic significance of S-TLS for 5-year OS. The presence of S-TLS was found to indicate a good prognosis only in LNM^–^ patients (*p* = 0.0232, Figure 4B) but not in the LNM^+^ patients (*p* = 0.1244, Figure 4C).

## 4. Discussion

In the present study, TLSs were classified according to previous studies in other tumors, which revealed that the presence of S-TLSs was an independent favorable prognostic factor for OS in patients with pCCA [24,32,33].

Recent successes of immune checkpoint inhibitors, such as anti-PD-1/PD-L1 and anti-CTLA-4, in a variety of human malignancies, have led to increased research interest in the function of the immune microenvironment during cholangiocarcinoma development and progression [38,39,40,41]. Additionally, a recent TOPAZ-1 clinical trial (Retrieved 6 December 2022, from ClinicalTrial.gov, NCT03875235) demonstrated that durvalumab can additionally improve the efficacy of chemotherapy in cholangiocarcinoma, demonstrating PD-L1 blockade’s potential as a first-line agent in advanced cholangiocarcinoma [13]. In recent years, increasing numbers of studies have shown that TLSs might create an important local microenvironment that encourages cellular and humoral immune responses targeted against cancer cells, and are believed to be favorable prognostic indicators for almost all solid human tumors [17,42]. Previous studies have shown that the prognostic significance of TLSs depends on their maturity. Compared with those tumors lacking GC-harboring TLSs (early/primary TLSs), tumors comprising mature GC-harboring TLSs (secondary TLSs) have a markedly reduced risk of recurrence [34]. Similarly, in chemotherapy-naïve patients with lung squamous cell carcinoma, only secondary TLSs were associated with improved survival, while early or primary TLSs were not [33].

In exploring the prognostic potential of the TLS populations with different levels of maturation, we divided the TLSs into lymphoid aggregates (Agg), primary follicle-like TLS (P-TLS), and secondary follicle-like TLS (S-TLS) according to their CD20^+^ B cell density and the follicular structures formed with GCs [24,32]. As mentioned above, pCCA patients were further divided into TLS^−^, Agg^+^, P-TLS^+^, and S-TLS^+^ according to the intra-tumoral TLS types. We found that although TLS^+^ patients with pCCA had a lower risk of recurrence, their OS was not significantly improved (Figure 3A,B). We also found significant improvement in the median OS and RFS in patients with S-TLS^+^ pCCA compared with patients with TLS^−^, Agg^+^, and P-TLS^+^ (Figure S1). Clearly, patient classification based on S-TLS status is a better predictor of recurrence and OS outcomes (Figure 3C,D). Given that S-TLSs typically comprise aggregates of CD20^+^ B cells and CD21^+^ FDCs, whereas Agg or P-TLS are not typical (Figure 2), the diverse prognosis in pCCA patients with different TLSs at different stages of maturity might be explained if FDCs promote long-term retention of the intact antigen in immune complexes and contribute to the survival of GC B cells and the maturation of GC affinity [43,44]. Moreover, the S-TLS status was the only independent prognostic factor for OS (not for recurrence) in our pCCA cohort (Table 3 and Supplementary Table S3). In the univariate analysis, we noted a poor prognosis of OS in patients with pCCA with lymph node metastasis and further speculated that the negative correlation between S-TLS and lymph node status might be partly responsible for the failed classification using lymph node status in multivariate analysis (Table 2 and Table 3), which suggested that S-TLS is a better predictor of OS than lymph node status in pCCA. Furthermore, our results found that S-TLS indicated a good prognosis only in LNM^−^ patients, not in LNM^+^ patients, suggesting a potential correlation between S-TLS and lymph node metastasis. This may be explained by the immune escape of tumor cells in LNM^+^ pCCA, which in turn leads to the loss of the antitumor function of S-TLS. These findings suggested that understanding and interpreting TLS composition, formation, and maturation in pCCA might facilitate targeted anti-cancer therapies and improve the poor survival outlooks of patients with pCCA.

Recent advances in cancer immunotherapy, for example, immune checkpoint inhibitors, have induced persistent responses in patients with a variety of cancers [45,46]. However, the largest proportion of patients does not respond to current immunotherapy treatments [47]. TLSs occur inside or next to tumor tissues, in which they enhance the infiltration and trafficking of lymphocytes, thus becoming a target to manipulate anti-tumor immunity [17,21,42,45]. Cancer-related TLS induction and manipulation might lead to novel strategies for tumor immunotherapy. To date, several TLS-targeted treatments have been reported, which demonstrated favorable anti-tumor effects in a variety of mouse models [46,47]. Alternatively, established cancer immunotherapies, such as anti-PD-1/PD-L1 and anti-CTLA-4, if used in combination with TLS neogenesis-inducing agents, might show enhanced effectiveness [48,49]. Considering the favorable prognosis associated with mature TLSs and the initial success of reagents promoting TLS formation in combination with existing immunotherapies in other tumors, drugs that can induce the production of mature TLS in tumors are expected to be a new option for anti-tumor immunotherapy of pCCA. However, clinical drugs that efficiently induce mature TLS have not yet been developed. Moreover, while our results suggest a predictive role for TLSs in pCCA outcomes, it also faces challenges in clinical applications, mainly because the acquisition of diagnostic specimens relies heavily on sampling techniques and the randomness of the puncture site, which may limit its wide use as a diagnostic and predictive marker. This bottleneck is expected to be broken in the future with the development of more and more improved puncturing techniques and their application to pCCA [50]. In future research, we aim to focus on the induction of pCCA-associated TLS induction in response to variations in cytokine and chemokine expression levels, combined with the use of appropriate biomaterials as a scaffold to boost the efficacy of anti-tumor immunity.

In summary, this study confirmed that intra-tumoral S-TLSs are present in human pCCA tissues, which was identified as a favorable prognostic indicator for OS in patients with pCCA. Our findings furnish a basis to better understand the tumor immune microenvironment of pCCA and are expected to provide new strategies for anti-tumor immunotherapy of pCCA.

## 5. Conclusions

Our results suggest that TLSs of varying maturity are present in pCCA tumor tissue and that only mature TLSs are considered favorable prognostic indicators of OS. In the present study, the potential role of TLS in pCCA is revealed for the first time, providing a basis for a comprehensive understanding of TLS’s role in pCCA. It also helps to deepen the understanding of the tumor immune microenvironment of pCCA and provides ideas for the generation of improved pCCA immunotherapy strategies.

## Figures and Tables

**Table 1:** Correlation analyses between intra-tumoral TLSs and the patients’ clinicopathological characteristics.

Clinicopathological Variables	Available Data (*n*)	Percentage (*n*%)	TLS^−^ pCCA *n* = 13 (14.0%)	TLS^+^ pCCA *n* = 80 (86.0%)	*p*-Value
Age, years					
≤60	54	58.06	10	44	0.137
>60	39	41.94	3	36	
Sex					
male	53	56.99	5	48	0.146
female	40	43.01	8	32	
Tumor size, cm					
≤2.5	27	29.03	2	25	0.333 *
>2.5	66	70.97	11	55	
Differentiation					
moderate + good	73	78.49	9	64	0.467 *
poor	20	21.51	4	16	
T stage					
T1 + T2	62	66.67	4	58	0.008 *
T3 + T4	31	33.33	9	22	
Lymph node metastasis					
absence	54	58.06	5	49	0.123
presence	39	41.94	8	31	
M stage					
M0	88	94.62	13	75	1 *
M1	5	5.38	0	5	
TNM stage					
I + II	39	41.94	2	37	0.036
III + IV	54	58.06	11	43	
CEA, ng/mL					
≤4	53	56.99	6	47	0.395
>4	40	43.01	7	33	
CA19-9, U/mL					
≤135	31	33.33	3	28	0.533 *
>135	62	66.67	10	52	
HBsAg					
absence	75	80.65	9	66	0.270 *
presence	18	19.35	4	14	
Microscopic residual tumor					
R0	69	74.19	9	60	0.735 *
R1	24	25.81	4	20	

TLS, tertiary lymphoid structure; CEA, carcinoembryonic antigen; CA19-9, carbohydrate antigen 19-9. * Fisher’s exact test was used, others Chi-squared test.

**Table 2:** Correlation analyses between intra-tumoral S-TLSs and the patients’ clinicopathological characteristics.

Clinicopathological Variables	S-TLS^−^ pCCA *n* = 32 (34.4%)	S-TLS^+^ pCCA *n* = 61 (65.6%)	*p*-Value
Age, years			
≤60	24	30	0.017
>60	8	31	
Sex			
male	14	39	0.062
female	18	22	
Tumor size, cm			
≤2.5	6	21	0.114
>2.5	26	40	
Differentiation			
moderate + good	24	49	0.552
poor	8	12	
T stage			
T1 + T2	17	45	0.045
T3 + T4	15	16	
Lymph node metastasis			
absence	12	42	0.004
presence	20	19	
M stage			
M0	31	57	0.657 *
M1	1	4	
TNM stage			
I + II	8	31	0.017
III + IV	24	30	
CEA, ng/mL			
≤4	17	36	0.586
>4	15	25	
CA19-9, U/mL			
≤135	8	23	0.217
>135	24	38	
HBsAg			
absence	25	50	0.656
presence	7	11	
Microscopic residual tumor			
R0	24	45	0.898
R1	8	16	

S-TLS, secondary follicle-like TLS; TLS, tertiary lymphoid structure; CEA, carcinoembryonic antigen; CA19-9, carbohydrate antigen 19-9. * Fisher’s exact test was used, others Chi-squared test.

**Table 3:** Univariate and multivariate analyses of overall survival.

Variables	OS					
	Univariate			Multivariate		
	HR	95% CI	*p*-Value	HR	95% CI	*p*-Value
Age, y (>60/≤60)	1.535	0.943–2.498	0.085			
Sex (male/female)	0.645	0.397–1.049	0.077			
Tumor size, cm (>2.5/≤2.5)	1.396	0.810–2.404	0.229			
Differentiation (poor/moderate + good)	1.628	0.918–2.888	0.095			
T stage (T3 + T4/T1 + T2)	1.381	0.836–2.281	0.207			
Lymph node metastasis (presence/absence)	1.870	1.151–3.038	0.012	1.291	0.745–2.237	0.363
M stage (M1/M0)	1.529	0.553–4.231	0.413			
TNM stage (III + IV/I + II)	1.585	0.956–2.626	0.074			
CEA, ng/mL (>4/≤4)	1.755	1.092–2.833	0.021	1.388	0.821–2.349	0.221
CA19-9, U/mL (>135/≤135)	1.850	1.112–3.049	0.016	1.768	0.975–3.205	0.061
HBsAg (presence/absence)	1.184	0.655–2.140	0.577			
Microscopic residual tumor (R1/R0)	1.141	0.674–1.932	0.623			
S-TLS (presence/absence)	0.472	0.284–0.784	0.004	0.570	0.330–0.985	0.044

Statistical analysis was performed using univariate and multivariate Cox proportional hazards regression models. TLS, tertiary lymphoid structure; S-TLS, secondary follicle-like TLS; CEA, carcinoembryonic antigen; CA19-9, carbohydrate antigen 19-9; HR, hazard ratio; OS, overall survival.

## Data Availability

All the data supporting the findings of this study are available within the article and its supplementary information files and available from the corresponding author upon reasonable request.

## Supplementary Materials

The following supporting information can be downloaded at: https://www.mdpi.com/article/10.3390/cancers14246107/s1, Figure S1: Impact of TLSs with different maturity levels on OS and RFS in patients with pCCA. Table S1: Correlation analyses between Agg and the patients’ clinicopathological characteristics; Table S2: Correlation analyses between P-TLS and the patients’ clinicopathological characteristics. Table S3: Univariate and multivariate analyses of pCCA recurrence.

## Abbreviations

CCA, Cholangiocarcinoma; pCCA, perihilar cholangiocarcinoma; dCCA, distal cholangiocarcinoma; iCCA, intrahepatic cholangiocarcinoma; TLS, tertiary lymphoid structures; S-TLS, secondary follicle-like TLS; P-TLS, primary follicle-like TLS; Agg, aggregate; RFS, Recurrence-free survival; OS, overall survival; PD-L1, programmed cell death 1 ligand 1; CTLA4, cytotoxic T-lymphocyte associated protein 4; HHLA2, Human Endogenous Retrovirus-H Long Terminal Repeat-Associating Protein 2; FGL1, fibrinogen Like 1; DC, dendritic cell; HEV, high endothelial venules; HCC, hepatocellular carcinoma; PNAD, protein N-terminal asparagine amidase; H&E, hematoxylin and eosin; FDC, follicular dendritic cell; GC, germinal centers; CEA, carcinoembryonic antigen; CA19-9, carbohydrate antigen 19-9.
