# Supplementary material for: Intra-Tumoral Secondary Follicle-like Tertiary Lymphoid Structures Are Associated with a Superior Prognosis of Overall Survival of Perihilar Cholangiocarcinoma
Source: Cancers (Basel). 2022 Dec 12;14(24):6107. doi: 10.3390/cancers14246107 (PMC9776022; doi:10.3390/cancers14246107)
Supplement: Supplementary file 1 [file cancers-14-06107-s001.zip › cancers-2075032-supplementary.pdf]

Intra-tumoral secondary follicle-like tertiary lymphoid structures are associated with a superior prognosis of overall survival of perihilar cholangiocarcinoma

Fa-Peng Zhang, Ke Zhu, Tai-Feng Zhu, Chao-Qun Liu, Hong-hua Zhang, Lei-bo Xu, Gang Xiao, Chao Liu

Table of contents

Supplementary Figure S1……………………………………………….2

Supplementary Table S1……………………………………………….3

Supplementary Table S2……………………………………………….4

Supplementary Table S3……………………………………………….5

Supplementary figure 1

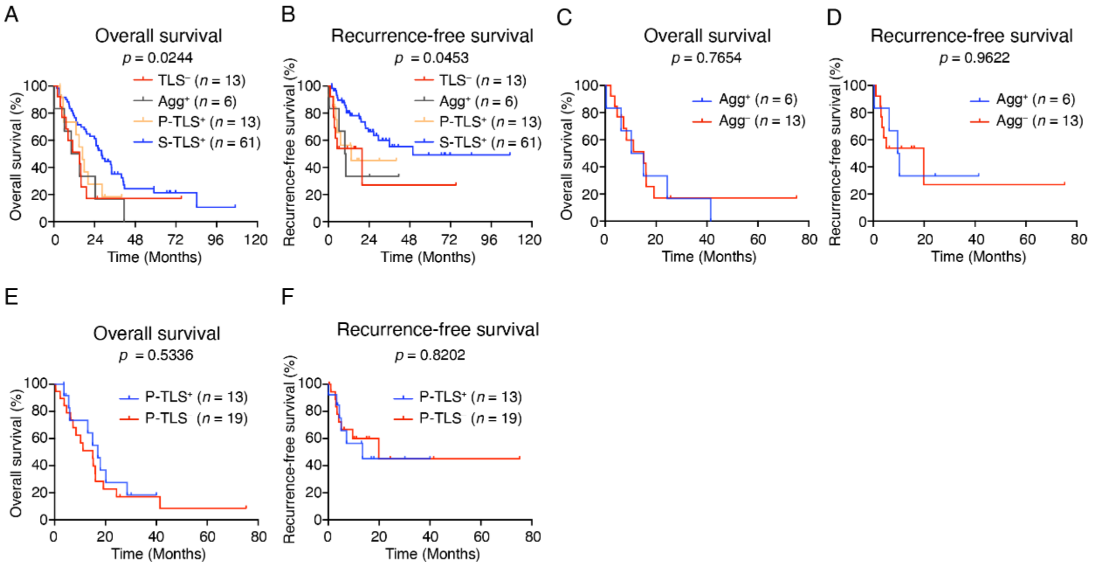

Supplemental Figure S1. Impact of TLSs with different maturity levels on OS and RFS in patients with pCCA. (A and B) Patients with intra-tumor S-TLSs had significantly improved OS and RFS compared with patients with TLS$^-$, Agg$^+$, and P-TLS$^+$ phenotypes; **(C–F)** Agg or P-TLS were not linked to OS and tumor relapse.

Supplementary Table S1. Correlation analyses between Agg and the patients' clinicopathological characteristics.

Clinicopathological Variables		Agg^{-} pCCA (n = 13)	Agg^{+} pCCA (n = 6)	*P* value
Age, years				
	≤ 60	10	3	0.320
	> 60	3	3	
Sex				
	male	5	3	1
	female	8	3	
Tumor size, cm				
	≤ 2.5	2	0	1
	> 2.5	11	6	
Differentiation				
	moderate + good	9	6	0.255
	poor	4	0	
T stage				
	T1 + T2	4	3	0.617
	T3+T4	9	3	
Lymph node metastasis				
	absence	5	2	1
	presence	8	4	
M stage				
	M0	13	6	NA
	M1	0	0	
TNM stage				
	I + II	2	1	1
	III + IV	11	5	
CEA, ng/ml				
	≤ 4	6	3	1
	> 4	7	3	
CA19-9, U/ml				
	≤ 250	3	2	1
	> 250	10	4	
HBsAg				
	absence	9	6	0.255
	presence	4	0	
Microscopic residual tumor				
	R0	9	4	1
	R1	4	2	

Agg, lymphoid aggregates; CEA, carcinoembryonic antigen; CA19-9, carbohydrate antigen 19-9. Statistical analysis was performed using Fisher's exact tests.

Supplementary Table S2. Correlation analyses between P-TLS and the patients' clinicopathological characteristics.

Clinicopathological Variables		P-TLS^{-} pCCA (n = 19)	P-TLS^{+} pCCA (n = 13)	*P* value
Age, years				
	≤ 60	13	11	0.420*
	> 60	6	2	
Sex				
	male	8	6	0.821
	female	11	7	
Tumor size, cm				
	≤ 2.5	2	4	0.194*
	> 2.5	17	9	
Differentiation				
	moderate + good	15	9	0.684*
	poor	4	4	
T stage				
	T1 + T2	7	10	0.026
	T3 + T4	12	3	
Lymph node metastasis				
	absence	7	5	1*
	presence	12	8	
M stage				
	M0	19	12	0.406*
	M1	0	1	
TNM stage				
	I + II	3	5	0.219*
	III + IV	16	8	
CEA, ng/ml				
	≤ 4	9	8	0.430
	> 4	10	5	
CA19-9, U/ml				
	≤ 135	5	3	1*
	> 135	14	10	
HBsAg				
	absence	15	10	1*
	presence	4	3	
Microscopic residual tumor				
	R0	13	11	0.420*
	R1	6	2	

P-TLS, primary follicle-like TLS; TLS, tertiary lymphoid structure; CEA, carcinoembryonic antigen; CA19-9, carbohydrate antigen 19-9.

* Fisher's exact test was used, others Chi-squared test.

Supplementary Table S3. Univariate and multivariate analyses of pCCA recurrence.

Variables	Recurrence					
	Univariate			Multivariate		
	HR	95% CI	*P* value	HR	95% CI	*P* value
Age, y (> 60/≤ 60)	1.034	0.522–2.047	0.923			
Sex (male/female)	0.433	0.221–0.848	0.015	0.473	0.239–0.936	0.031
Tumor size, cm (> 2.5/≤ 2.5)	1.22	0.584–2.546	0.597			
Differentiation (poor/moderate + good)	0.728	0.281–1.884	0.513			
T stage (T3 + T4/T1 + T2)	1.924	0.984–3.764	0.056			
Lymph node metastasis (presence/absence)	1.811	0.928–3.535	0.082			
M stage (M1/M0)	0.046	0–38.676	0.37			
TNM stage (III + IV/I + II)	1.994	0.975–4.077	0.059			
CEA, ng/ml (> 4/≤ 4)	2.257	1.155–4.412	0.017	1.891	0.934–3.827	0.077
CA19-9, U/ml (> 135/≤ 135)	2.806	1.223–6.44	0.015	1.993	0.830–4.783	0.123
HBsAg (presence/absence)	0.889	0.369–2.146	0.794			
Microscopic residual tumor (R1/R0)	1.312	0.642–2.682	0.456			
S-TLS (presence/absence)	0.477	0.24–0.949	0.035	0.586	0.29–1.184	0.137

Statistical analysis was performed using univariate and multivariate Cox proportional hazards regression models.

TLS, tertiary lymphoid structure; S-TLS, secondary follicle-like TLS; CEA, carcinoembryonic antigen; CA19-9, carbohydrate antigen 19-9; HR, hazard ratio.
